# Deficiency of the BMP Type I receptor ALK3 partly protects mice from anemia of inflammation

**DOI:** 10.1186/s12899-018-0037-z

**Published:** 2018-02-27

**Authors:** Inka Gallitz, Niklas Lofruthe, Lisa Traeger, Nicole Bäumer, Verena Hoerr, Cornelius Faber, Tanja Kuhlmann, Carsten Müller-Tidow, Andrea U. Steinbicker

**Affiliations:** 1 Department of Anaesthesiology, Intensive Care and Pain Medicine, University Hospital Muenster, Albert-Schweitzer Campus 1, Building A1, 48149 Muenster, Germany; 2 Department of Medicine A, Molecular Haematology and Oncology, University Hospital Muenster, 48149 Muenster, Germany; 3 Institute of Medical Microbiology, Jena University Hospital, 07747 Jena, Germany; 4 Department of Clinical Radiology, University Hospital Muenster, 48149 Muenster, Germany; 5 Institute for Neuropathology, University Hospital Muenster, 48149 Muenster, Germany; 6 Present Address: Department of Medicine V, Hematology, Oncology and Rheumatology, Heidelberg University Hospital, 69120 Heidelberg, Germany

**Keywords:** Bone Morphogenetic Protein (BMP) type I receptor, Inflammation, Iron, Hepcidin, Liver

## Abstract

**Background:**

Inflammatory stimuli induce the hepatic iron regulatory hormone hepcidin, which contributes to anaemia of inflammation (AI). Hepcidin expression is regulated by the bone morphogenetic protein (BMP) and the interleukin-6 (IL-6) signalling pathways. Prior results indicate that the BMP type I receptor ALK3 is mainly involved in the acute inflammatory hepcidin induction four and 72 h after IL-6 administration. In this study, the role of ALK3 in a chronic model of inflammation was investigated. The intact, heat-killed bacterium *Brucella abortus* (BA) was used to analyse its effect on the development of inflammation and hypoferremia in mice with hepatocyte-specific *Alk3*-deficiency (*Alk3*^fl/fl^; *Alb-Cre*) compared to control (*Alk3*^fl/fl^) mice.

**Results:**

An iron restricted diet prevented development of the iron overload phenotype in mice with hepatocyte-specific *Alk3* deficiency. Regular diet leads to iron overload and increased haemoglobin levels in these mice, which protects from the development of AI per se. Fourteen days after BA injection *Alk3*^fl/fl^; *Alb-Cre* mice presented milder anaemia (Hb 16.7 g/dl to 11.6 g/dl) compared to *Alk3*^fl/fl^ control mice (Hb 14.9 g/dl to 8.6 g/dl). BA injection led to an intact inflammatory response in all groups of mice. In *Alk3*^fl/fl^; *Alb-Cre* mice, SMAD1/5/8 phosphorylation was reduced after BA as well as after infection with *Staphylococcus aureus.* The reduction of the SMAD1/5/8 signalling pathway due to hepatocyte-specific *Alk3* deficiency partly suppressed the induction of STAT3 signalling.

**Conclusion:**

The results reveal in vivo, that 1) hepatocyte-specific *Alk3* deficiency partly protects from AI, 2) the development of hypoferremia is partly dependent on ALK3, and 3) the ALK3/BMP/hepcidin axis may serve as a possible therapeutic target to attenuate AI.

**Electronic supplementary material:**

The online version of this article (10.1186/s12899-018-0037-z) contains supplementary material, which is available to authorized users.

## Background

Patients with acute or chronic inflammatory diseases or malignancies often develop anaemia of inflammation (AI). More than a quarter of the world’s population is anaemic. As the second most common form of anaemia, AI contributes substantially to the burden of disease [1, 2]. Patients with AI present with hypoferremia, as iron is trapped within the iron stores and iron bioavailability for erythropoiesis is therefore decreased [3]. The hepatic hormone hepcidin plays a major role in the maintenance of iron homeostasis. Hepcidin induces internalization and degradation of the sole known iron exporter ferroportin [4]. Induction of hepcidin decreases the bioavailability of iron and can lead to an iron-restricted erythropoiesis [5]. During inflammation, cytokines such as the pro-inflammatory cytokine interleukin 6 (IL-6) are induced [6]. IL-6 acts via the Janus kinase-signal transducer (JAK) and activator of transcription (STAT3) pathway. Hepcidin transcription is induced by binding of phosphorylated STAT3 to the STAT3-responsive element in the hepcidin promoter [7]. In addition, transcriptional regulation of hepcidin requires BMP signalling [8]. Upon ligand binding, the BMP type II receptor is phosphorylated and thereby activates the BMP type I receptor. Activation of the kinase domain of the BMP type I receptor phosphorylates SMAD (P-SMAD 1, 5, and 8) proteins, which translocate with SMAD4 to the nucleus. Hepcidin expression is induced after binding of the P-SMAD complex to the BMP-responsive element in the hepcidin promoter [9, 10]. Specific inhibitors of the BMP signalling pathway present novel therapeutic opportunities in different diseases such as pulmonary arterial hypertension, vascular calcification or AI [11, 12]. The hepatocyte-specific disruption of SMAD4, or hepatocyte-specific deficiency of the BMP type I receptors ALK2 or ALK3, cause moderate and severe iron overload and hepcidin suppression, respectively [9, 13]. Mice with hepatocyte-specific *Smad4* deficiency display not only attenuated baseline hepcidin expression, but also lack IL-6 mediated hepcidin expression [9]. In mice with hepatocyte-specific *Alk*3 deficiency, neither iron nor BMP agonists were able to stimulate hepcidin [13]. In a model of acute inflammation, Mayeur et al. demonstrated, that IL-6 induced hepatic hepcidin mRNA expression after four (or 72) hours was dependent on the BMP type I receptor ALK3 [14]. In mice with hepatocyte-specific *Alk3* deficiency STAT3 was phosphorylated by IL-6, but the hepcidin induction was impeded and hepcidin levels remained at about 1–5% compared to control mice in short term experiments [13, 14]. The susceptibility of these mice to develop AI had yet to be investigated. The intraperitoneal application of a single dose of heat-killed *Brucella abortus* (BA) particles was utilized in this study to induce chronic inflammation and the development of AI in mice. This well described murine model of AI features the following hallmarks of the disease: i) early hepcidin induction, ii) cytokine release and, iii) impaired erythropoiesis [15–17].

We hypothesized that suppressed hepcidin levels and iron overload in hepatocyte-specific *Alk3* deficiency protect from AI development per se. Prior to BA or saline injection and throughout the experiment, nutritional iron was therefore restricted in all groups of mice in order to maintain similar baseline iron levels in mice with and without hepatocyte-specific *Alk3* deficiency. In a previous study, nutritional iron restriction did not alter the early BA mediated induction of hepatic hepcidin mRNA levels or BA mediated serum IL-6 induction in control mice [18]. As anaemia suppresses hepcidin expression per se*,* and Kim et al. reported decreased hepcidin expression 14 days after BA administration in WT mice due to anaemia, STAT3 and SMAD1/5/8 phosphorylation were investigated in a second model [16] *: S. aureus* was applied to control and hepatocyte-specific *Alk3* deficient mice on a standard rodent chow and proteins were analysed 24 h later. Upon *S. aureus* administration SMAD1/5/8 phosphorylation was detectable in control mice, but not in mice with hepatocyte-specific *Alk3* deficiency.

To conclude, various factors contribute to anaemia. The presented data indicate that ALK3 and subsequently hepcidin are involved in the cross-talk between iron and inflammation, and contribute to at least 30% of the AI development in this model. Therefore, ALK3 inhibition could be an approach to ameliorate AI.

## Methods

### Animal research

Mice with homozygous loxP-flanked (“floxed”) Alk3 alleles (*Alk3*^fl/fl^) on a C57BL/6 background were bred with B6.Cg-Tg^(Alb-Cre)21Mgn^/J mice (Jackson Laboratory) to obtain homozygous animals (*Alk3*^fl/fl^) with or without the hepatocyte-specific *Cre* recombinase driven by an albumin promotor [19]. Mice with hepatocyte-specific deficiency of *Alk3* (*Alk3*^fl/fl^; *Alb-Cre*) were compared to *Alk3*^fl/fl^ mice without expression of the *Alb-Cre* as described previously (on regular iron chow) [13]*.* In this study, all mice were fed an iron-deficient diet since weaning and throughout the experiment (5 ppm iron, Altromin C1038, Lage, Germany).

### Murine heat-killed *Brucella abortus* model and injection with *Staphylococcus aureus*

All mouse experiments were carried out in accordance with the recommendations and approval of the institutional ethics committee for “Animal Care of North Rhine-Westphalia, the Landesamt fuer Natur, Umwelt und Verbraucherschutz (LANUV), North Rhine-Westphalia, Germany” permit numbers LANUV Az.84–02.04.2013.A281 and 87–51.04.2011.A003. *Brucella abortus* (BA, Strain 99, *Brucella abortus* MRT AG PA 0048) was prepared as described by Sasu et al. [17]. Mice were maintained according to institutional guidelines in individually ventilated cages and were given food and water ad libitum. 12-week-old female mice were injected once with BA (5 × 10^8^ particles per mouse) or PBS intraperitoneally (Additional file 1a). Fourteen days after BA administration, mice had an average weight of 24 g ± 2,9. At that day blood withdrawal and organ collection were performed, when Hb levels reach nadir values [16]. Mice were sacrificed by cervical dislocation in deep anaesthesia.

*Staphylococcus aureus* (*S. aureus*) strain 6850 (ATCC 53657, Manassas, VA) was cultivated overnight in brain-heart infusion medium under shaking conditions at 37 °C [20]. The bacteria were washed twice with sterile PBS and the bacterial suspension was adjusted to optical density at 600 nm (OD600 = 1), and stored at − 80 °C until use. Mice were inoculated with 1 × 10^6^ colony forming units (CFUs) of *S. aureus* microorganisms in 150 μL of PBS or with PBS alone as vehicle via a lateral tail vein. After 24 h, blood was collected in deep anaesthesia (Additional file 1b). Then mice were sacrificed by cervical dislocation and organs were collected. All animals were monitored daily. No sudden deaths occurred.

### Erythroid progenitor cells

Bone marrow (BM) was collected and processed as described previously [21]. Cells were stained with APC-conjugated rat-anti-mouse CD44 (BD Pharmingen, Heidelberg, Germany) and PE-conjugated rat-anti-mouse TER119 (BD Pharmingen, Heidelberg, Germany) in 2% FBS/PBS for 30 min at RT protected from light. Analysis was performed using the BD FACSDiva™ software on a FACSCalibur™ (Becton Dickinson, Heidelberg, Germany). Unstained cells were used as negative controls. Mean fluorescence intensity (FLI) from 20.000 cell counts was used as a measure of protein surface expression of Ter119 and CD44 [22–24].

### Reticulocytes

Reticulocytes were counted by flow cytometry (FACSCalibur™, Becton Dickinson, Heidelberg, Germany). Blood (5 μl) was added to 1 ml of thiazole orange reagent (Retic-COUNT™, BD Bioscience; San Jose, CA) and incubated at room temperature for 1 h. Unstained controls were used to establish a gate to exclude background fluorescence. The results are expressed as the reticulocyte production index: RPI = Retic% x Hb/14.46, with 14.46 g/dL as the mean baseline haemoglobin (Hb) level of healthy WT mice [18].

### Hematologic and iron parameters

Blood samples were collected by retro-orbital puncture and serum iron parameters were determined as previously described [13]. Complete blood counts were obtained with the scil Vet abc Plus™ (Viernheim, Germany). Non-haem tissue iron levels were determined as previously described [25].

### Hepatic mRNA levels

RNA was extracted from tissue using Trizol® (Sigma, Hamburg, Germany) and homogenized with an ultrasound dissector. cDNA was created by MMLV-reverse transcriptase (Sigma, Hamburg, Germany).

Quantitative RT-PCR was performed on an Applied Biosystems 7500 Fast Real-Time-PCR system with LuminoCt® SYBR® Green qPCR ReadyMix™ (Sigma, Hamburg, Germany). The relative CT method was used to normalize the levels of target transcripts to 18S rRNA levels (Additional file 2).

### Protein analysis

Liver tissue samples were prepared with RIPA buffer supplemented with protease and phosphatase inhibitors (Sigma-Aldrich, Heidelberg, Germany). Extracted proteins (40 μg/lane) were separated by electrophoresis using 4%–10% bis-tris gels and nitrocellulose membranes (GE Healthcare, Freiburg, Germany). Membranes were incubated with antibodies directed against phosphorylated STAT3 (at tyrosine705 (P-STAT3, Cat. No. 9145 L, Cell Signalling Technology)), STAT3 (Cat. No. 4904, Cell Signalling Technology), phosphorylated SMAD 1/5/8 (P-SMAD 1/5, Cat. No. 9516S, Cell Signalling Technology), SMAD1 (Cat. No. 6944, Cell Signalling Technology), Ferroportin (Cat. No. MTP11-A, Alpha Diagnostics) and α-tubulin (Cat. No. T6074, Sigma-Aldrich). Washed membranes were incubated with horseradish peroxidase–linked anti-rabbit or anti-mouse IgG (New England Biolabs, Frankfurt, Germany). Membranes were incubated with ECL-Plus (Bio-Rad, Munich, Germany), and chemiluminescence was detected with a ChemiDoc™ XRS+ (Bio-Rad, Munich, Germany). Densitometrical analysis was performed with Image Lab™ (Bio-Rad, Munich Germany).

### Statistical analysis

All values are expressed as mean ± SD. Data were analysed using nonparametric Mann-Whitney U test with two tailed *P* values. Differences were considered statistically significant with *P* ≤ 0.05 (*).

### Data availability

The data supporting the conclusions of this article are included within the article and the Additional file 3.

## Results

### Hepatocyte-specific deficiency of *Alk3* attenuated AI development

**Fig. 1:**
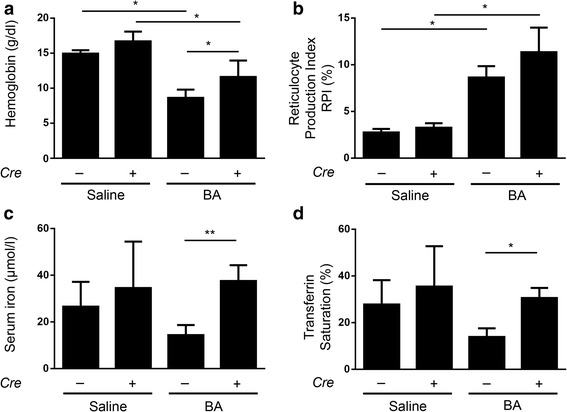
Milder Anaemia of inflammation in *Alk3*^fl/fl^; *Alb-Cre* mice compared to *Alk3*^fl/fl^ mice. **a** Haemoglobin values from *Alk3*^fl/fl^ and *Alk3*^fl/fl^; *Alb-Cre* mice 14 days after heat-killed *Brucella abortus* (BA) or saline injection (*P = 0.01: *Alk3*^fl/fl^ injected with saline [*n* = 4] vs *Alk3*^fl/fl^ injected with BA [*n* = 6]; *P = 0.01: *Alk3*^fl/fl^; *Alb-Cre* injected with saline [*n* = 4] vs *Alk3*^fl/fl^; *Alb-Cre* injected with BA [*n* = 6]; *P = 0.04: *Alk3*^fl/fl^ injected with BA [*n* = 6] vs *Alk3*^fl/fl^; *Alb-Cre* injected with BA [*n* = 6]). **b** Reticulocyte Production Index (*P = 0.03: *Alk3*^fl/fl^ injected with saline vs *Alk3*^fl/fl^ injected with BA; *P = 0.01: *Alk3*^fl/fl^; *Alb-Cre* injected with saline vs *Alk3*^fl/fl^; *Alb-Cre* injected with BA). **c** Serum iron from *Alk3*^fl/fl^ and *Alk3*^fl/fl^; *Alb-Cre* mice 14 days after BA administration (**P = 0.004: *Alk3*^fl/fl^ injected with BA vs *Alk3*^fl/fl^; *Alb-Cre* injected with BA). **d** Transferrin saturation (**P = 0.01: *Alk3*^fl/fl^ injected with BA vs *Alk3*^fl/fl^; *Alb-Cre* injected with BA [*n* = 5])

Mice with hepatocyte-specific *Alk3* deficiency fed a standard rodent diet develop iron overload. Iron overload could protect these mice from anaemia. In order to avoid development of the iron overload phenotype, mice were fed an iron–deficient diet since weaning and throughout the experiment. At the age of 12 weeks mice were exposed to the heat-killed *BA* model of AI. Alk3 mRNA levels were suppressed in *Alk3*^fl/fl^; *Alb-Cre* mice injected either with saline or with BA, and compared to *Alk3*^fl/fl^ control mice (Additional file 4). Fed an iron-deficient diet, *Alk3*^fl/fl^; *Alb-Cre* and *Alk3*^fl/fl^ control mice injected with PBS expressed comparable Hb levels, reticulocyte production index (RPI), serum iron levels and transferrin saturation (Fig. 1a-d). Tissue iron content in liver and spleen (Additional file 5) was comparable between control and hepatocyte-specific *Alk3* deficient mice. BA administration led to a slight increase in LIC of hepatocyte-specific *Alk3* deficient mice compared to saline application in these mice. The results indicate that development of the iron overload phenotype in *Alk3*^fl/fl^; *Alb-Cre* compared to *Alk3*^fl/fl^ mice was prevented by the iron-deficient diet. Fourteen days after the intraperitoneal injection of BA*, Alk3*^fl/fl^ control mice developed anaemia indicated by a decrease of the mean Hb levels from 14.9 g/dl to 8.6 g/dl (Fig. 1a). In contrast, *Alk3*^fl/fl^; *Alb-Cre* mice presented moderate anaemia with a decrease in Hb levels from 16.7 g/dl to 11.6 g/dl. As a description of the relative reduction, Hb levels in control mice after BA administration were about one third lower compared to *Alk3* deficient mice after BA administration. The data indicate that mice with hepatocyte-specific *Alk3* deficiency were partially protected from the development of AI and hypoferremia.

**Fig. 2:**
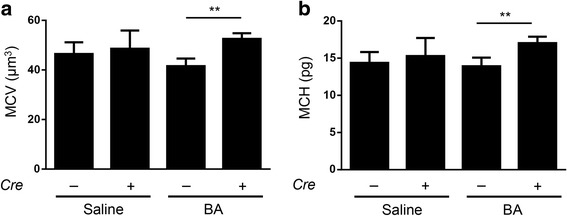
MCV and MCH levels in *Alk3*^fl/fl^; *Alb-Cre* mice compared to *Alk3*^fl/fl^ mice. **a** Mean corpuscular volume (MCV) (**P = 0.002: *Alk3*^fl/fl^ injected with BA vs *Alk3*^fl/fl^; *Alb-Cre* injected with BA), and **b** Mean corpuscular haemoglobin (MCH) (**P = 0.002: *Alk3*^fl/fl^ injected with BA vs *Alk3*^fl/fl^; *Alb-Cre* injected with BA)

The RPI was increased in both groups after BA administration (Fig. 1b) due to anaemia. Fed an iron-deficient diet and injected with PBS, *Alk3*^fl/fl^; *Alb-Cre* mice and *Alk3*^fl/fl^ mice presented comparable serum iron levels and transferrin saturation (Fig. 1c-d). Fourteen days after BA injection *Alk3*^fl/fl^; *Alb-Cre* mice showed higher serum iron levels and higher transferrin saturation compared to BA treated *Alk3*^fl/fl^ mice (Fig. 1c-d). In particular, the mean corpuscular volume (MCV) and the mean corpuscular haemoglobin (MCH) values were higher in *Alk3*^fl/fl^; *Alb-Cre* mice injected with BA than in *Alk3*^fl/fl^ after BA injection (Fig. 2a-b). While *Alk3*^fl/fl^ mice fed an iron deficient diet and injected with BA developed a microcytic, hypochromic AI, the *Alk3*^fl/fl^; *Alb-Cre* mice treated equally, developed a normocytic, normochromic AI with normal MCV and MCH values (Fig. 2a-b).

**Fig. 3:**
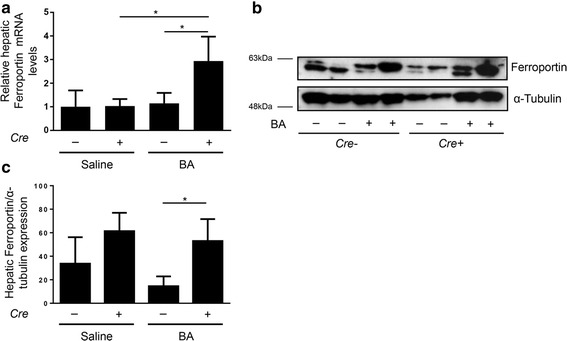
Hepatocyte-specific *Alk3* deficiency resulted in elevated liver ferroportin expression 14 days after BA injection. **a** Relative hepatic ferroportin mRNA levels (*P = 0.01: *Alk3*^fl/fl^; *Alb-Cre* injected with saline [*n* = 4] vs *Alk3*^fl/fl^; *Alb-Cre* injected with BA [*n* = 6]; *P = 0.01: *Alk3*^fl/fl^ injected with BA [*n* = 5] vs *Alk3*^fl/fl^; *Alb-Cre* injected with BA [*n* = 6]). Representative Western blots (**b**) and quantitative analyses (**c**) of hepatic ferroportin protein levels with α-tubulin as loading control

**Fig. 4:**
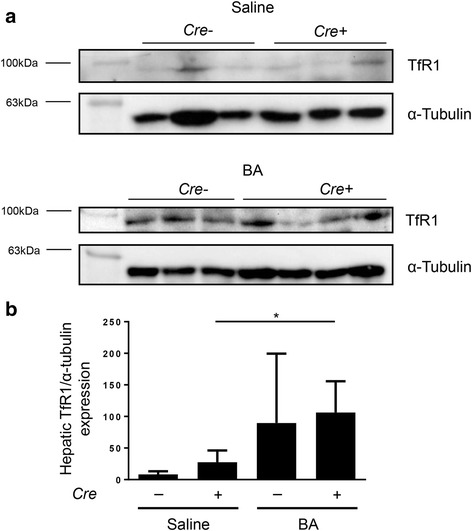
Hepatic protein levels of transferrin receptor 1. **a** Representative Western blots and quantitative analyses (**b**) of hepatic TfR1 protein levels in *Alk3*^fl/fl^ and *Alk3*^fl/fl^; *Alb-Cre* mice 14 days after heat-killed *Brucella abortus* (BA) injection. As loading control α-tubulin expression is depicted. (*P = 0.03: *Alk3*^fl/fl^; *Alb-Cre* injected with saline vs *Alk3*^fl/fl^; *Alb-Cre* injected with BA)

In mice with hepatocyte-specific *Alk*3 deficiency BA induced hepatic ferroportin mRNA levels. Protein levels remained similarly high due to the hepcidin reduction caused by hepatocyte-specific *Alk3* deficiency (Fig. 3a-c). Hepatic ferritin expression was induced after BA application in control and in mice with hepatocyte-specific *Alk3* deficiency (Additional file 6). Hepatic TfR1 expression was elevated in hepatocyte-specific *Alk3* deficient mice after BA application. In control mice, a similar trend was observed (Fig. 4a-b). Splenic ferroportin mRNA levels were induced by BA in both, control and mice with hepatocyte-specific *Alk3* deficiency (Additional file 7). Splenic TfR1 mRNA levels were only elevated in control mice after BA administration (Additional file 7).

These data indicate that the BA-mediated decrease in serum iron levels led to an elevated extramedullary erythropoiesis as indicated by increased hepatic TfR1 protein and splenic TfR1 mRNA levels in *Alk3*^*fl/fl*^*; Alb-Cre* and *Alk3*^*fl/fl*^ mice, respectively. Ferritin is not only an iron storage marker, but also an acute phase protein. Therefore, hepatic ferritin expression was induced after BA application in control and in mice with hepatocyte-specific *Alk3* deficiency as part of the acute phase reaction. LICs were slightly increased (Additional files 5 and 6).

There were no discernible differences detectable in mRNA levels of ferroportin in the duodenum (Additional file 7). However, immunofluorescence staining of duodenal ferroportin of untreated mice with and without hepatocyte-specific *Alk3* deficiency indicated a stronger ferroportin expression on the luminal surface of the mucosa in mice with hepatocyte-specific *Alk3* deficiency compared to control mice (Additional files 8 and 9). These data suggest that a regional difference in ferroportin expression may result in the better iron mobilization in mice with hepatocyte-specific *Alk3* deficiency.

Taken together, the data indicate that mice with hepatocyte-specific deficiency of *Alk3* under iron-restricted conditions still developed AI after BA administration, but to a milder extent than control mice. Due to the relative reduction in haemoglobin levels, the contribution of the ALK3/hepcidin/ferroportin circuitry to BA-mediated AI was estimated with about 30%. Hepatocyte-specific *Alk3* deficiency partly prevented the development of hypoferremia and led to normocytic, normochromic erythrocytes.

### The inflammatory response to BA administration was intact in mice with hepatocyte-specific *Alk3* deficiency

**Fig. 5:**
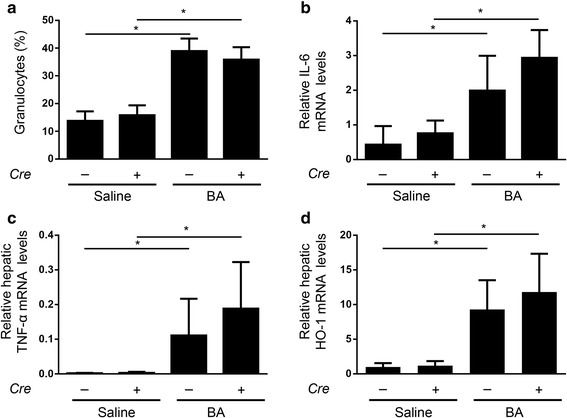
Comparable BA-induced inflammation in *Alk3*^fl/fl^; *Alb-Cre* mice and *Alk3*^fl/fl^ mice. **a** Granulocytes from *Alk3*^fl/fl^ and *Alk3*^fl/fl^; *Alb-Cre* mice 14 days after heat-killed *Brucella abortus* (BA) or saline injection (*P = 0.01: *Alk3*^fl/fl^ injected with saline [*n* = 4] vs *Alk3*^fl/fl^ injected with BA [*n* = 4]; *P = 0.01: *Alk3*^fl/fl^; *Alb-Cre* injected with saline [*n* = 4] vs *Alk3*^fl/fl^; *Alb-Cre* injected with BA [*n* = 6]), and (**b**) Relative hepatic IL-6 mRNA levels from *Alk3*^fl/fl^ and *Alk3*^fl/fl^; *Alb-Cre* mice 14 days after BA administration (*P = 0.03: *Alk3*^fl/fl^ injected with saline [*n* = 3] vs *Alk3*^fl/fl^ injected with BA [*n* = 5]; **P = 0.01: *Alk3*^fl/fl^; *Alb-Cre* injected with saline [*n* = 4] vs *Alk3*^fl/fl^; *Alb-Cre* injected with BA [*n* = 6]). **c** Relative hepatic TNF-α mRNA levels (*P = 0.04: *Alk3*^fl/fl^ injected with saline [*n* = 3] vs *Alk3*^fl/fl^ injected with BA [*n* = 4]; *P = 0.01: *Alk3*^fl/fl^; *Alb-Cre* injected with saline [*n* = 5] vs *Alk3*^fl/fl^; *Alb-Cre* injected with BA [*n* = 6]). **d** Relative hepatic HO-1 mRNA levels (*P = 0.01: *Alk3*^fl/fl^ injected with saline [*n* = 4] vs *Alk3*^fl/fl^ injected with BA [*n* = 6]; *P = 0.01: *Alk3*^fl/fl^; *Alb-Cre* injected with saline [*n* = 4] vs *Alk3*^fl/fl^; *Alb-Cre* injected with BA [*n* = 6])

**Fig. 6:**
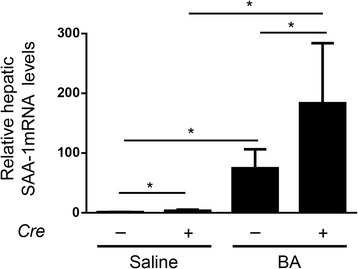
BA-induced SAA-1 mRNA levels in *Alk3*^fl/fl^; *Alb-Cre* mice and *Alk3*^fl/fl^ mice. Relative hepatic SAA-1 mRNA levels (*P = 0,01: *Alk3*^fl/fl^ injected with saline [*n* = 4] vs *Alk3*^fl/fl^ injected with BA [*n* = 4]; *P = 0,01: *Alk3*^fl/fl^; *Alb-Cre* injected with saline [*n* = 6] vs *Alk3*^fl/fl^; *Alb-Cre* injected with BA [*n* = 6]; *P = 0.03: *Alk3*^fl/fl^ injected with saline vs *Alk3*^fl/fl^; *Alb-Cre* injected with saline; ^*^P = 0.03: *Alk3*^fl/fl^ injected with BA vs *Alk3*^fl/fl^; *Alb-Cre* injected with BA). The relative CT method was used to normalize the levels of target transcripts to 18S rRNA levels

In order to determine the inflammatory response to BA, mice with and without hepatocyte-specific *Alk3* deficiency were analysed 14d after BA injection. AI was accompanied by an induction of granulocytes within comparable ranges after BA application in *Alk3*^fl/fl^ and *Alk3*^fl/fl^; *Alb-Cre* mice (Fig. 5a), which indicate comparable grades of chronic inflammation in both groups. IL-6 and TNF-α mRNA levels were induced in both, *Alk3*^fl/fl^; *Alb-Cre* and *Alk3*^fl/fl^ mice, to a similar extent (Fig. 5b-c). HO-1 mRNA expression levels, a marker of oxidative stress, which is upregulated by the IL-6/STAT3 signalling pathway, were elevated in the liver of both, *Alk3*^fl/fl^ and *Alk3*^fl/fl^; *Alb-Cre* mice, after BA administration (Fig. 5d). IL-6 induces SAA-1 mRNA levels. Therefore, BA administration led to an increase in SAA-1 mRNA in *Alk3*^fl/fl^; *Alb-Cre* and *Alk3*^fl/fl^ mice (Fig. 6). Interestingly, SAA-1 mRNA levels were markedly higher in *Alk3*^fl/fl^; *Alb-Cre* mice compared to *Alk3*^fl/fl^ mice injected with BA. Hepcidin mRNA levels were suppressed 14d after BA-injection in all four groups of mice (Additional file 10). These data were expected, as the BA injection causes a hepcidin increase after 6 h, which decreases in chronic, prolonged inflammation and iron deficiency after 14d. To conclude, the BA-mediated inflammatory response was not altered in mice with hepatocyte-specific *Alk3* deficiency. The elevated SAA-1 levels in mice with hepatocyte-specific *Alk3* deficiency might reflect the failure of early hepcidin induction upon inflammation and its anti-inflammatory properties.

### Erythropoiesis was similar in *Alk3*^fl/fl^ and *Alk3*^fl/fl^; *Alb-Cre* mice after BA administration

**Fig. 7:**
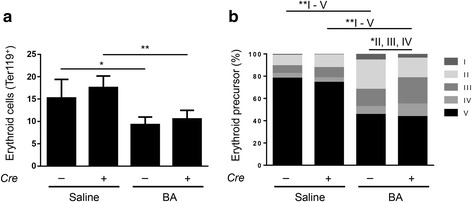
Bone marrow erythropoiesis depicted through total erythroid cells and distribution of erythroid precursor cell populations. **a** Total erythroid cells (Ter119+) in the bone marrow 14 days after heat-killed *Brucella abortus* (BA) or saline injection (*P = 0.03: *Alk3*^fl/fl^ injected with saline [*n* = 4] vs *Alk3*^fl/fl^ injected with BA [*n* = 6]; **P = 0.009: *Alk3*^fl/fl^; *Alb-Cre* injected with saline [*n* = 4] vs *Alk3*^fl/fl^; *Alb-Cre* injected with BA [*n* = 6]). **b** Erythroid subpopulations in the bone marrow of *Alk3*^fl/fl^ and *Alk3*^fl/fl^; *Alb-Cre* mice 14d after BA administration (V and II: *P = 0.01: *Alk3*^fl/fl^ injected with saline vs *Alk3*^fl/fl^ injected with BA; III: *P = 0.01: *Alk3*^fl/fl^ injected with saline vs *Alk3*^fl/fl^ injected with BA; III and V: *P = 0.01: *Alk3*^fl/fl^; *Alb-Cre* injected with saline vs *Alk3*^fl/fl^; *Alb-Cre* injected with BA, II: *P* = 0.01: *Alk3*^fl/fl^ injected with BA vs *Alk3*^fl/fl^; *Alb-Cre* injected with BA; III: *P* = 0.01: *Alk3*^fl/fl^ injected with BA vs *Alk3*^fl/fl^; *Alb-Cre* injected with BA). [Subpopulation V = terminal differentiated red cells, IV = orthochromatic erythroblast, III = polychromatic erythroblast, II = basophilic erythroblast, I = proerythroblast]

To investigate whether an impaired erythropoiesis contributed to AI, total BM cells were selected for Ter119 cell surface expression via fluorescence-activated cell sorter to identify the amount of erythroid precursor cells. Cells from *Alk3*^fl/fl^ and *Alk3*^fl/fl^; *Alb-Cre* mice exhibited a similar reduction in their total erythroid cell population (43% decrease in *Alk3*^fl/fl^ mice compared with 39% decrease in *Alk3*^fl/fl^; *Alb-Cre* mice. Ter119^+^ cells, Fig. 7a). To further distinguish the erythroid subpopulations, cells were sorted by CD44 expression and cell size (forward scatter). In later, further differentiated stages of erythroblasts, the CD44 surface expression declines (from proerythroblast to reticulocyte).

Gating for the Terr119^+^ cell population, the subpopulations were analysed. BA administration led to an upregulation in basophilic erythroblasts in *Alk3*^fl/fl^ mice only (population II, Fig. 7b). Polychromatic erythroblasts (population III) were upregulated in both groups, but more pronounced in *Alk3*^fl/fl^; *Alb-Cre* mice. BA administration resulted in a comparable decrease of terminal differentiated red cells from the BM in both groups (terminal differentiated red cells, population V, Fig. 7b). However, despite the differences in erythroid precursor cell distribution between both groups, BA administration led to an arrest in the maturation of erythroid cells before they differentiated into orthochromatic erythroblasts. This resulted in equally suppressed numbers of terminal differentiated red cells (population V) and the total of erythroid cells (Ter119^+^) from the BM in both, *Alk3*^fl/fl^ mice and *Alk3*^fl/fl^; *Alb-Cre* mice. Additionally, the number of red blood cells (RBCs) was equally decreased in both groups after BA administration (Additional file 10). The data reflect that BM erythropoiesis did not contribute to the protection of BA-mediated AI in *Alk3*^fl/fl^; *Alb-Cre* mice compared to controls. BA administration resulted in an impaired erythropoiesis of the BM in both, *Alk3*^fl/fl^ and *Alk3*^fl/fl^; *Alb-Cre* mice, with an arrest in maturation prior to differentiation into orthochromatic erythroblasts. The ratio of splenic weight/body weight was comparable and indicate that both groups compensated for the impaired erythropoiesis of the BM after BA administration with an enhanced erythropoiesis in the spleen (Additional file 10). The suppression of hepcidin develops due to the secretion of erythroid factors, such as erythroferrone, by the bone marrow. The way these erythroid factors repress hepcidin expression is partly known: Wang et al. showed that SMAD1/5 was required for erythropoietin and erythroferrone mediated hepcidin suppression [26]. As erythroferrone is proximal of BMP/SMAD signalling, there was no difference in erythroferrone mRNA in liver and spleen of mice with and without hepatocyte-specific *Alk3* deficiency (Additional file 10).

### Inhibition of BMP signalling did affect SMAD1/5/8 phosphorylation

**Fig. 8:**
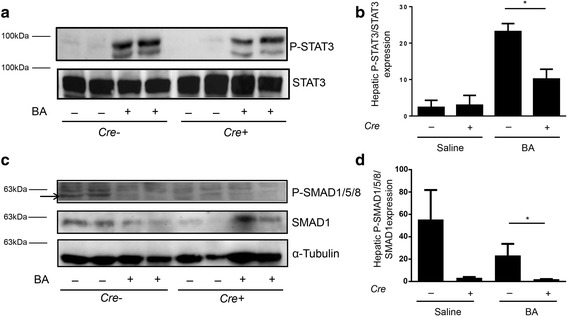
Hepatic STAT3 and SMAD1/5/8 protein expression in *Alk3*^fl/fl^; *Alb-Cre* and *Alk3*^fl/fl^ mice. Representative western blots (**a**) and quantitative analyses (**b**) of phospho-STAT3 and total-STAT3 from *Alk3*^fl/fl^ and *Alk3*^fl/fl^; *Alb-Cre* mice 14 days after heat-killed *Brucella abortus* (BA) or saline injection**.** Two isoforms of phospho-STAT3 α and β (79/86 kDa) exist. Loading control is indicated by total STAT3 protein levels. **c** SMAD1/5/8 activation 14d after BA administration in *Alk3*^fl/fl^ (*Cre-*) and hepatocyte-specific *Alk3* deficient (*Alk3*^fl/fl^; *Alb-Cre*) (*Cre+*) mice fed an iron deficient diet. Western blots from liver proteins analysed with phosphorylated SMAD1/5/8, total SMAD1, and α-tubulin antibodies. Arrows indicate specific pSMAD signal. **d** Densitometric analyses of the ratio of SMAD1/5/8 to SMAD1

In order to determine, if STAT3 phosphorylation was intact in mice with and without hepatocyte-specific *Alk3* deficiency, STAT3 phosphorylation was determined via immunoblotting. BA-injection induces IL-6 and thereby STAT3 phosphorylation. STAT3 phosphorylation was present in *Alk3*^fl/fl^ and *Alk3*^fl/fl^; *Alb-Cre* mice injected with BA, but weaker in the latter (Fig. 8a-b). SMAD1/5/8 baseline phosphorylation was detectable in control, but not in *Alk3* deficient mice. Fourteen days after BA administration SMAD1/5/8 phosphorylation seems reduced in control mice and still absent in *Alk3* deficient mice (Fig. 8c-d). These results indicate that BMP signalling is abrogated in mice with hepatocyte-specific *Alk3* deficiency, so that ALK3 is the critical receptor for intact BMP/SMAD signalling and that SMAD activation is required for the STAT3 pathway.

**Fig. 9:**
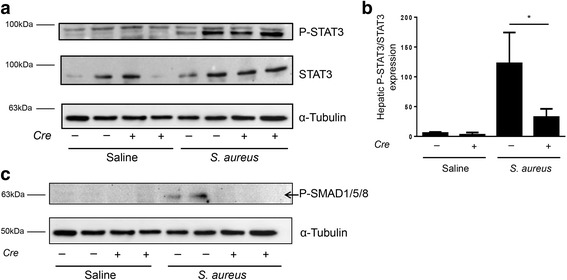
Hepatic protein levels of STAT3 and SMAD1/5/8. Representative western blots (**a**) and quantitative analyses (**b**) of hepatic phospho-STAT3 and total-STAT3 protein expression from *Alk3*^fl/fl^ (*Cre-*) and *Alk3*^fl/fl^; *Alb-Cre* (*Cre+*) mice 24 h after *S. aureus* administration. Two isoforms of phospho-STAT3 α and β (79/86 kDa) exist. Loading control is depicted by total STAT3 protein levels. **c** Hepatic protein expression of phospho-SMAD1/5/8, SMAD1, and α-tubulin from *Alk3*^fl/fl^ (*Cre-*) and *Alk3*^fl/fl^; *Alb-Cre* (*Cre+*) mice 24 h after *S. aureus* administration

As SMAD1/5/8 phosphorylation, due to iron restriction and anaemia, was not induced 14 days after heat-killed BA administration, we administered the vital bacterium *S. aureus* in another set of experiments to mice with and without hepatocyte-specific *Alk3* deficiency and analysed the protein phosphorylation 24 h later. In mice with and without hepatocyte-specific *Alk3* deficiency, STAT3 phosphorylation was detectable 24 h after the *S. aureus* injection (Fig. 9a-b). SMAD1/5/8 phosphorylation was only detectable in control mice 24 h after *S. aureus* administration (Fig. 9c).

To conclude, the inhibition of the SMAD1/5/8 signalling pathway due to *Alk3* deficiency partly suppressed the induction of the STAT3 signalling pathway by BA or *S. aureus* administration.

## Discussion

AI is the second most common form of anaemia worldwide. Pathophysiologically, AI is associated with elevated cytokine levels, iron trapped in iron stores, iron restricted erythropoiesis and hypoferremia. The current manuscript reveals for the first time that hepatocyte-specific *Alk3* deficiency partly protects mice from development of AI. The iron restricted diet fed prior to the experiment prevented development of the iron overload phenotype in *Alk3*^fl/fl^; *Alb-Cre* mice. After BA injection, these *Alk3*^fl/fl^; *Alb-Cre* mice presented higher Hb levels, serum iron levels, transferrin saturation, MCV, and MCH compared to BA injected control mice. In contrast, the inflammatory response to BA administration was not altered by hepatocyte-specific *Alk3* deficiency as indicated by comparably elevated granulocytes and cytokine levels. BM erythropoiesis was equally suppressed in control and *Alk3*^fl/fl^; *Alb-Cre* mice due to elevated cytokine levels. The data indicate that ALK3 was required for the cytokine mediated development of hypoferremia. The ALK3-hepcidin axis accounted with about one third to the development of AI. Therefore, ALK3 serves as a possible therapeutic target for AI.

Of note, the BA model used in this study cannot be compared to active bacterial infection, as heat-killed *Brucella abortus* bacteria do not replicate. Nevertheless, it is a well-known and frequently used model for AI that was previously used to investigate AI by Kim, Sasu, Kautz, Gardenghi and stated in a review by Fraenkel [15–17, 27, 28]. The typical pathophysiological development that leads to AI, an induction of hepcidin, is also caused by the BA injection after 6 h independent from iron restricted diet [16, 18].

The peptide hormone hepcidin has been reported to play an important role in development of AI [29]. Mice lacking hepcidin (*Hamp*-KO) fed an iron-deficient diet showed milder anaemia and faster recovery after BA administration compared to control mice [15, 16]. The IL-6/hepcidin signalling pathway plays a major role in the development of anaemia in an inflammatory condition. Steinbicker et al. demonstrated that the hepatocyte-specific deficiency of the BMP type I receptor ALK3 not only led to a suppression of basal hepcidin expression and iron overload (mice on a regular diet), but that ALK3 was required for the hepatic hepcidin mRNA induction by iron, BMP ligands and IL-6 [13, 30]. Based upon these findings, we investigated the effect of the hepatocyte-specific *Alk3* deficiency on the development of AI in a well described mouse model for AI [15–17]. Even with an abolished iron overload phenotype in *Alk3*^fl/fl^; *Alb-Cre* mice fed an iron-restricted diet the hepatocyte-specific *Alk3* deficiency had a partially protective effect on the development of anaemia. The BMP type I receptor–SMAD-hepcidin signalling pathway contributed - as described in this manuscript - with about 30% to the development of anaemia in *Alk3*^fl/fl^; *Alb-Cre* mice. Kim et al. and Gardenghi et al. reported for *Hamp-*KO and *IL-6-*KO mice similar findings with a protection of about 30% to the development of BA-induced anaemia [15, 16]. Pan et al. reported that *Smad4*-deficient mice developed severe anaemia with a decrease in their Hb levels by 70% compared to WT mice, which was not only due to hepcidin suppression in the liver, but due to blood loss caused by polyps in the stomach and colon of these mice [31]. *Alk3*^fl/fl^; *Alb-Cre* mice displayed higher serum iron, Hb, transferrin saturation, MCV, and MCH levels compared to control mice after BA administration despite the iron-deficient diet.

This indicates that the lack of hepatic hepcidin expression in *Alk3*^fl/fl^; *Alb-Cre* mice led to alterations in iron metabolism. An enhanced haemoglobinisation of RBCs contributed to the partial protection from anaemia.

Production of pro-inflammatory cytokines in inflammation triggers the development of anaemia via induction of hepcidin. In *Alk3*^fl/fl^; *Alb-Cre* mice the induction of hepcidin was inhibited. BA administration led to an up-regulation of hepatic IL-6, TNF-α, and HO-1 mRNA levels in both, *Alk3*^fl/fl^ and *Alk3*^fl/fl^; *Alb-Cre* mice, and higher SAA-1 mRNA levels in the latter. Granulocytosis indicates a chronic inflammation in both control and *Alk3* deficient mice. These data indicate that the inflammatory response was intact in both groups. The lack of early cytokine-induced hepcidin expression (after 6 h) might have caused the induction of SAA-1 mRNA levels in mice with hepatocyte-specific *Alk3* deficiency. In erythroferrone-deficient mice Kautz et al. not only observed a more severe AI upon BA administration (due to a lack of hepcidin suppression), but also lower SAA-1 levels accompanied by higher hepcidin levels [27]. Additionally, Pagani et al. observed higher SAA-1 levels after LPS administration in mice with iron-deficiency [32]. Pre-treatment with hepcidin or high serum levels such as in *Tmprss6*^−/−^ mice led to blunted inflammatory responses (and lower SAA-1 levels) [32]. Wang et al. reported that mice with hepatocyte-specific *Smad4* deficiency show a lack of hepcidin induction and display elevated SAA-1 levels upon IL-6 administration [9]. The results indicate that the lack of hepcidin and not iron deficiency per se accounted for the elevated SAA-1 levels. These results are in line with the data presented in the current manuscript.

As expected, BA or *S. aureus* administration did not induce SMAD1/5/8 phosphorylation in mice with hepatocyte-specific *Alk3* deficiency. In control mice, *S. aureus*, but not BA application, led to an induction of SMAD1/5/8 phosphorylation. This might be due to the pronounced anaemia 14d after BA application. STAT3 phosphorylation was induced in control mice after BA or *S. aureus* administration. In mice with hepatocyte-specific *Alk3* deficiency, the induction was lower compared to *Alk3*^*fl/fl*^ mice. The data indicate that STAT3 phosphorylation requires intact BMP signalling.

The elevated hepatic ferroportin level and the protection from hypoferremia in hepatocyte-specific *Alk3* deficient mice indicate that the blunted BMP/SMAD signalling modulated the iron status in AI.

These results are in line with Ferga-Falzacappa et al., who determined the essential role of the SMAD binding element of the hepcidin promoter for hepcidin induction [10]. Furthermore Steinbicker et al. demonstrated that inhibition with the BMP type I receptor inhibitor LDN-193189 intraperitoneally, treated AI in wild-type mice [30]. Mayeur et al. reported that LDN-193189 given orally at a dose of 1 mg/kg to WT mice partially treated turpentine induced anaemia [33]. Taken together, the novel data of in vivo experiments of chronic inflammation in mice with and without hepatocyte-specific *Alk3* deficiency exposed to the BA and *S. aureus* model underline that ALK3 is the dominant BMP type I receptor of iron regulation in inflammation.

## Conclusion

The current manuscript revealed in vivo for the first time that 1.) the previously described iron overload phenotype of hepatocyte-specific *Alk3* deficient mice could be blunted by iron restricted diet and, 2.) hepatocyte-specific *Alk3* deficient mice were protected against development of severe AI. The results of the chronic model of inflammation and AI support the findings of short term exposure to IL-6 in these mice, published previously [14]. ALK3 was essentially required for IL-6 and BA mediated hepcidin induction. As hypothesized previously in cell culture studies and short term experiments, the BA-AI in vivo experiment of chronic inflammation revealed that the BMP pathway with the dominant receptor ALK3 is essentially required for intact hepcidin induction by inflammation, the development of hypoferremia, and partly for the development of AI. The inflammatory IL-6-STAT3 pathway should not be seen as an independent pathway- it depends on an intact BMP pathway.

## Additional files

Additional file 1: Experimental design. (a) Mice were fed an iron deficient diet since weaning and throughout the experiment. At the age of 12 weeks, female *Alk3*^fl/fl^; *Alb-Cre* and Alk3^fl/fl^ mice were intraperitoneally injected with 5 × 10^8^ particles/mouse of heat-killed *Brucella abortus* (BA) or saline. Two weeks later blood and organs were collected. (b) 12 week old *Alk3*^fl/fl^; *Alb-Cre* and *Alk3*^fl/fl^ female mice fed a regular diet were intravenously inoculated with 1 × 10^6^ colony forming units (CFUs) of *Staphylococcus aureus*. Twenty-four hours later blood and organs were collected. (TIFF 164 kb)

Additional file 2: **Table S1**. Semi-quantitative real-time PCR primer. (DOCX 15 kb)

Additional file 3: Raw data and analysis of the presented study. (XLSX 18 kb)

Additional file 4: Hepatocyte-specific *Alk3* deficiency resulted in suppressed liver Alk3 and hepcidin mRNA expression. Relative hepatic *Alk3* mRNA levels from *Alk3*^fl/fl^ and *Alk3*^fl/fl^; *Alb-Cre* 14 days after heat-killed *Brucella abortus* (BA) injection (*P = 0.03: *Alk3*^fl/fl^ injected with saline [*n* = 4] vs *Alk3*^fl/fl^; *Alb-Cre* saline [*n* = 4]; **P = 0.004: *Alk3*^fl/fl^ injected with BA [*n* = 6] vs *Alk3*^fl/fl^; *Alb-Cre* injected with BA [*n* = 6]. (TIFF 65 kb)

Additional file 5: Liver and spleen iron content from *Alk3*^fl/fl^ and *Alk3*^fl/fl^; *Alb-Cre* mice 14 days after BA challenge. (a) Liver iron content in *Alk3*^fl/fl^ and *Alk3*^fl/fl^; *Alb-Cre* mice 14 days after heat-killed *Brucella abortus* (BA) injection (*P = 0.04: *Alk3*^fl/fl^; *Alb-Cre* injected with saline [*n* = 4] vs *Alk3*^fl/fl^; *Alb-Cre* injected with BA [*n* = 6]). (b) Spleen iron content from *Alk3*^fl/fl^ and *Alk3*^fl/fl^; *Alb-Cre* mice 14 days after heat-killed *Brucella abortus* (BA) injection. (TIFF 82 kb)

Additional file 6: Hepatic ferritin expression in *Alk3*^fl/fl^ and *Alk3*^fl/fl^;*Alb-Cre* mice 14 days after BA challenge. Representative western blots (a) and quantitative analyses (b) of hepatic ferritin protein levels in *Alk3*^fl/fl^ and *Alk3*^fl/fl^; *Alb-Cre* mice 14 days after heat-killed *Brucella abortus* (BA) injection. As loading control α-tubulin expression is depicted. (TIFF 270 kb)

Additional file 7: Spleen and duodenum mRNA levels in *Alk3*^fl/fl^ and *Alk3*^fl/fl^;*Alb-Cre* mice 14 days after BA challenge. (a) Relative splenic ferroportin mRNA levels from *Alk3*^fl/fl^ and *Alk3*^fl/fl^; *Alb-Cre* 14 days after heat-killed *Brucella abortus* (BA) injection (*P = 0.02: *Alk3*^fl/fl^ injected with saline [*n* = 4] vs *Alk3*^fl/fl^ injected with BA [*n* = 5]; *P = 0.03: *Alk3*^fl/fl^; *Alb-Cre* injected with saline [*n* = 4] vs *Alk3*^fl/fl^; *Alb-Cre* injected with BA [*n* = 4]. (b) Relative splenic TfR1 mRNA levels (*P = 0.01: *Alk3*^fl/fl^ injected with saline [*n* = 4] vs *Alk3*^fl/fl^ injected with BA [*n* = 6]). (c) Relative duodenal ferroportin mRNA levels. The relative CT method was used to normalize the levels of target transcripts to 18S rRNA levels. (TIFF 103 kb)

Additional file 8: Immunofluorescence staining of ferroportin in the duodenum of *Alk3*^fl/fl^ and *Alk3*^fl/fl^; *Alb-Cre* mice*.* Ferroportin immunofluorescence staining of formalin fixed paraffin sections of the villosities of the duodenum with 20 times magnification. (Panel a) Control mice with nuclear (DAPI) and ferroportin (FITC) staining of a duodenal section. Cutout images merged (nuclear and ferroportin) and ferroportin (FITC) alone. (Panel b) Duodenal section of hepatocyte-specific *Alk3* deficient mice with cutout images merged (nuclear and ferroportin) and ferroportin (FITC) alone. White arrows highlight specific FPN staining. (TIFF 3601 kb)

Additional file 9: Supporting material and references. (DOCX 14 kb)

Additional file 10: Hepatic hepcidin mRNA levels, red blood cell count, spleen to bodyweight ratio, and hepatic erfe mRNA levels of *Alk3*^fl/fl^; *Alb-Cre* mice and *Alk3*^fl/fl^ mice. (a) Relative hepatic hepcidin mRNA levels compared to control mice fed a regular diet. The relative CT method was used to normalize the levels of target transcripts to 18S mRNA levels. (b) Red blood cell count from *Alk3*^fl/fl^ and *Alk3*^fl/fl^; *Alb-Cre* mice 14 days after heat-killed *Brucella abortus* (BA) injection (*P = 0.01: *Alk3*^fl/fl^ injected with saline [*n* = 4] vs *Alk3*^fl/fl^ injected with BA [*n* = 6]; *P = 0.02: *Alk3*^fl/fl^; *Alb-Cre* injected with saline [*n* = 4] vs *Alk3*^fl/fl^; *Alb-Cre* injected with BA [*n* = 6]). (c) Spleen to bodyweight ratio in *Alk3*^fl/fl^ and *Alk3*^fl/fl^; *Alb-Cre* mice 14 days after heat-killed *Brucella abortus* (BA) injection (*P = 0.01: *Alk3*^fl/fl^ injected with saline [*n* = 4] vs *Alk3*^fl/fl^ injected with BA [*n* = 6]; *P = 0.01: *Alk3*^fl/fl^; *Alb-Cre* injected with saline [*n* = 4] vs *Alk3*^fl/fl^; *Alb-Cre* injected with BA [*n* = 6]). (d) Relative hepatic erfe mRNA levels of *Alk3*^fl/fl^ and *Alk3*^fl/fl^; *Alb-Cre* mice 14 days after heat-killed *Brucella abortus* (BA) injection (*P = 0.01: *Alk3*^fl/fl^; *Alb-Cre* injected with saline [*n* = 4] vs *Alk3*^fl/fl^; *Alb-Cre* injected with BA [*n* = 5]). (TIFF 129 kb)

## Abbreviations

AI: Anaemia of inflammation
Alk3: Activin receptor-like kinase
BMP: Bone morphogenetic protein
Cre: Cyclization recombination
Hb: Haemoglobin
IL: Interleukin
MCH: Mean corpuscular haemoglobin
MCV: Mean corpuscular volume
mRNA: Messenger RNA
SMAD: Small Mothers Against Decapentaplegic homolog
STAT: Signal transducer and activator of transcription
TNF-α: Tumor necrosis factor α
