# Supplementary figures and images for: Deficiency of the BMP Type I receptor ALK3 partly protects mice from anemia of inflammation
Source: BMC Physiol. 2018 Feb 27;18:3. doi: 10.1186/s12899-018-0037-z (PMC6389079; doi:10.1186/s12899-018-0037-z)

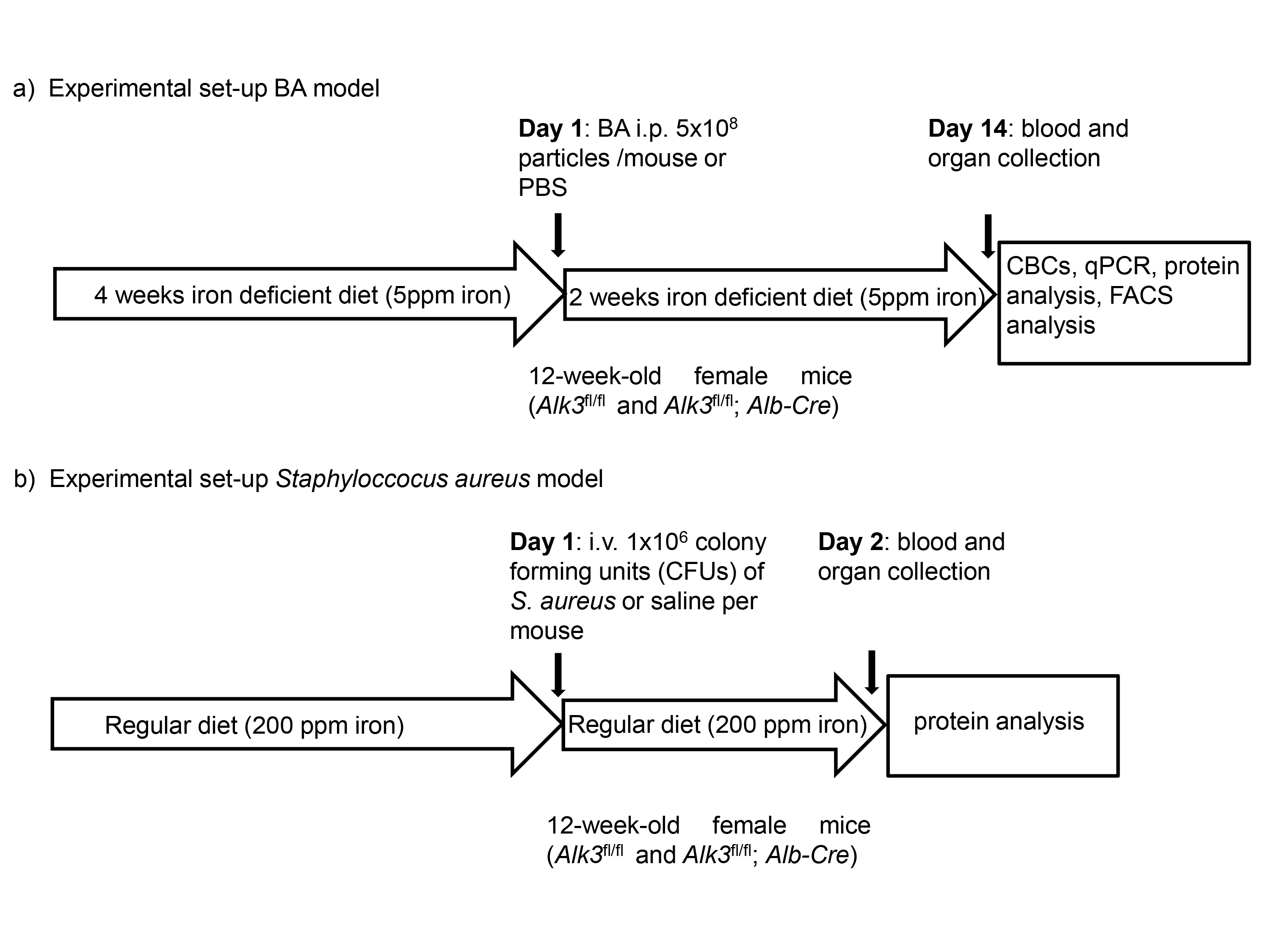

Supplement: Supplementary file 1 — Experimental design. (a) Mice were fed an iron deficient diet since weaning and throughout the experiment. At the age of 12 weeks, female Alk3fl/fl; Alb-Cre and Alk3fl/fl mice were intraperitoneally injected with 5 × 10^8^ particles/mouse of heat-killed Brucella abortus (BA) or saline. Two weeks later blood and organs were collected. (b) 12 week old Alk3fl/fl; Alb-Cre and Alk3fl/fl female mice fed a regular diet were intravenously inoculated with 1 × 10^6^ colony forming units (CFUs) of Staphylococcus aureus. Twenty-four hours later blood and organs were collected. (TIFF 164 kb) [file 12899_2018_37_MOESM1_ESM.tif]

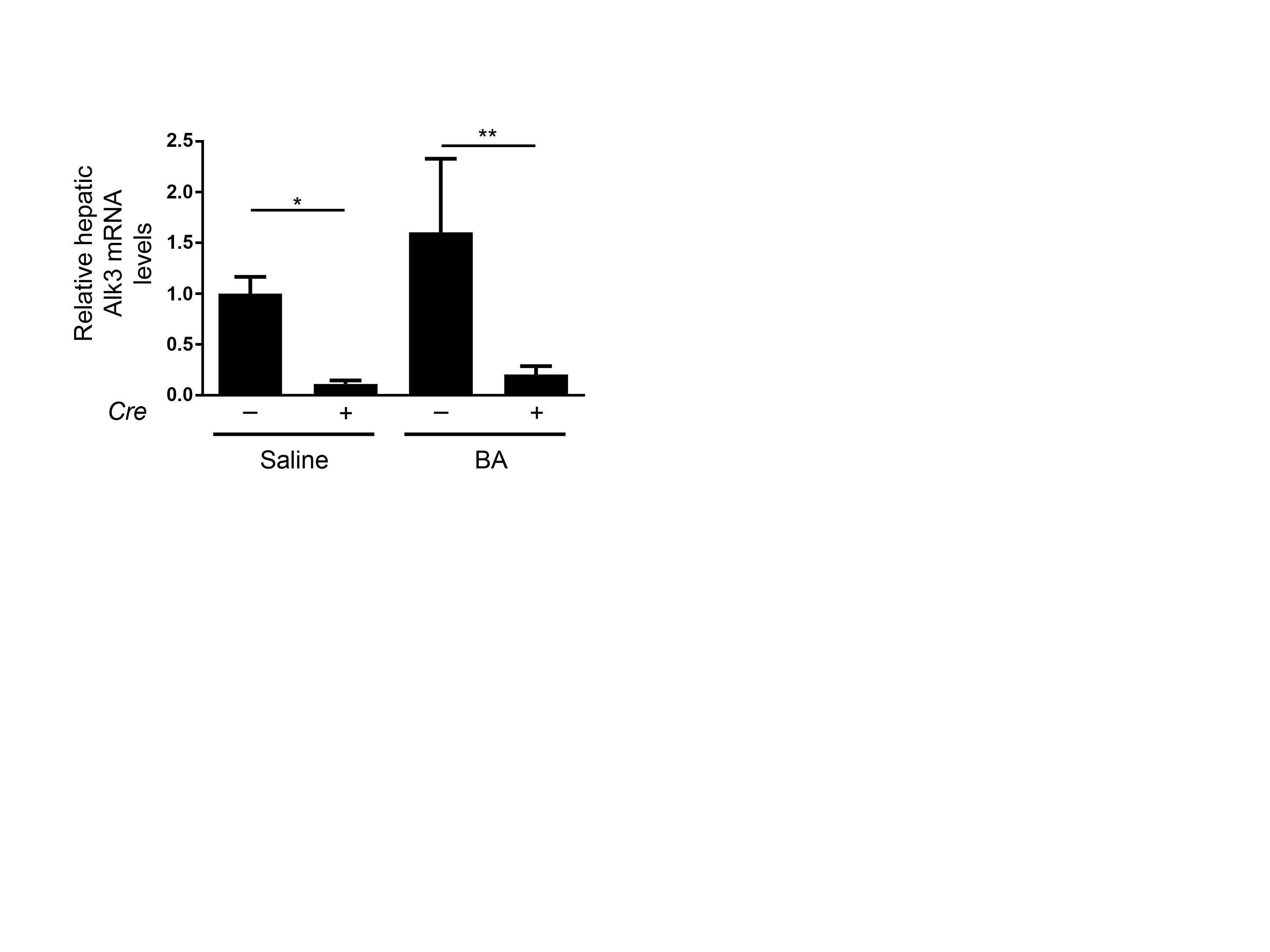

Supplement: Supplementary file 4 — Hepatocyte-specific Alk3 deficiency resulted in suppressed liver Alk3 and hepcidin mRNA expression. Relative hepatic Alk3 mRNA levels from Alk3fl/fl and Alk3fl/fl; Alb-Cre 14 days after heat-killed Brucella abortus (BA) injection (*P = 0.03: Alk3fl/fl injected with saline [n = 4] vs Alk3fl/fl; Alb-Cre saline [n = 4]; **P = 0.004: Alk3fl/fl injected with BA [n = 6] vs Alk3fl/fl; Alb-Cre injected with BA [n = 6]. (TIFF 65 kb) [file 12899_2018_37_MOESM4_ESM.tif]

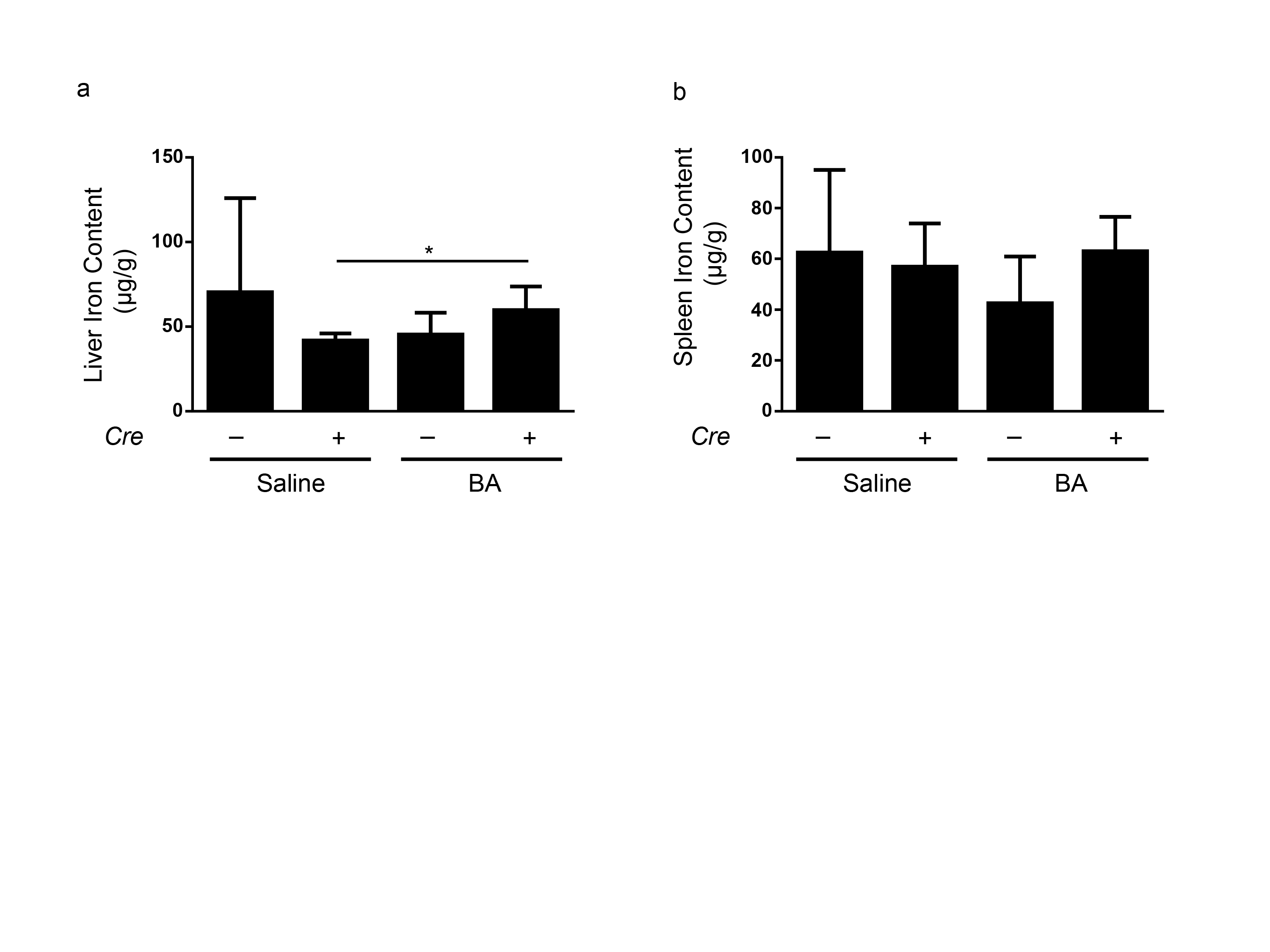

Supplement: Supplementary file 5 — Liver and spleen iron content from Alk3fl/fl and Alk3fl/fl; Alb-Cre mice 14 days after BA challenge. (a) Liver iron content in Alk3fl/fl and Alk3fl/fl; Alb-Cre mice 14 days after heat-killed Brucella abortus (BA) injection (*P = 0.04: Alk3fl/fl; Alb-Cre injected with saline [n = 4] vs Alk3fl/fl; Alb-Cre injected with BA [n = 6]). (b) Spleen iron content from Alk3fl/fl and Alk3fl/fl; Alb-Cre mice 14 days after heat-killed Brucella abortus (BA) injection. (TIFF 82 kb) [file 12899_2018_37_MOESM5_ESM.tif]

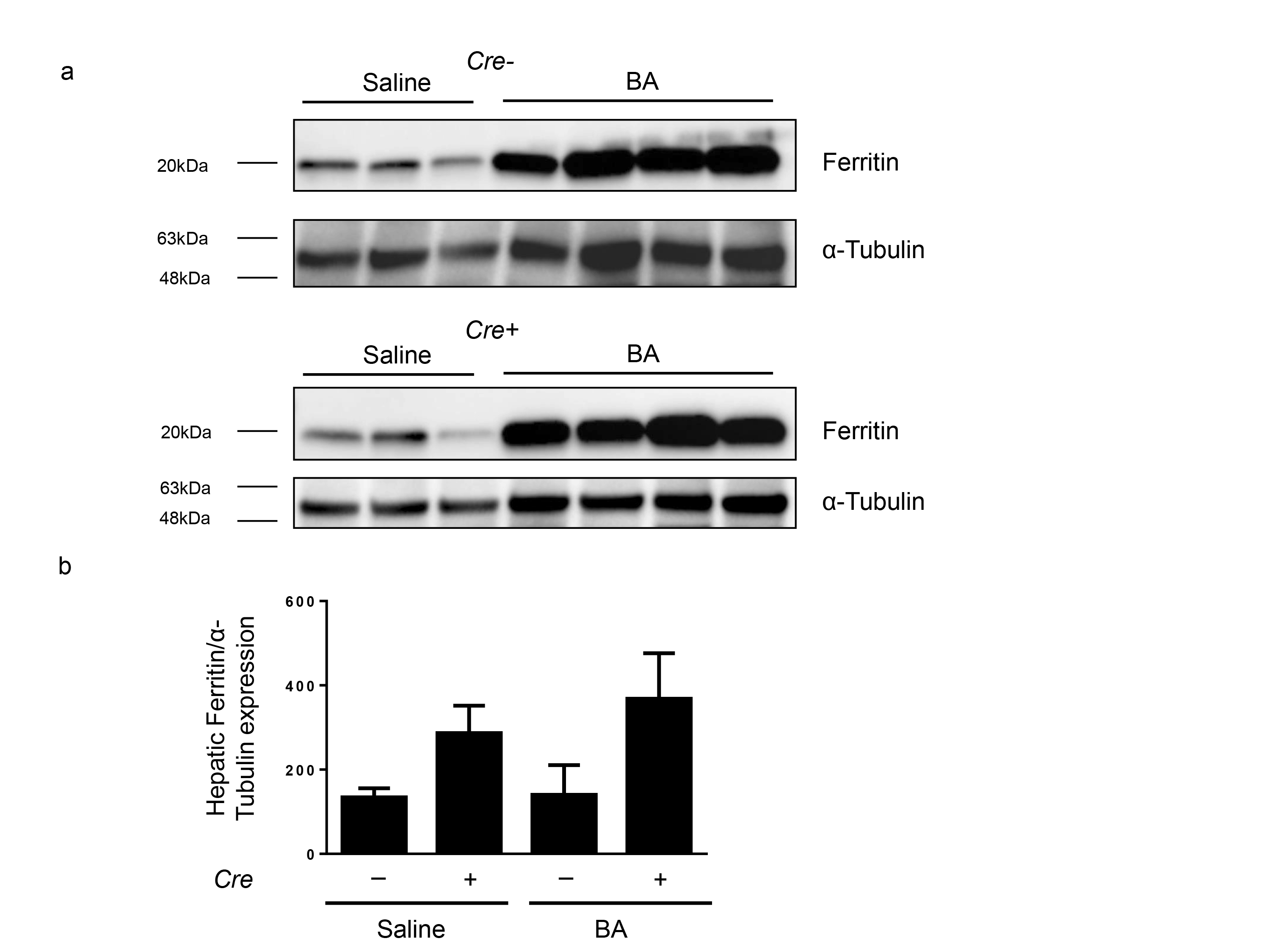

Supplement: Supplementary file 6 — Hepatic ferritin expression in Alk3fl/fl and Alk3fl/fl;Alb-Cre mice 14 days after BA challenge. Representative western blots (a) and quantitative analyses (b) of hepatic ferritin protein levels in Alk3fl/fl and Alk3fl/fl; Alb-Cre mice 14 days after heat-killed Brucella abortus (BA) injection. As loading control α-tubulin expression is depicted. (TIFF 270 kb) [file 12899_2018_37_MOESM6_ESM.tif]

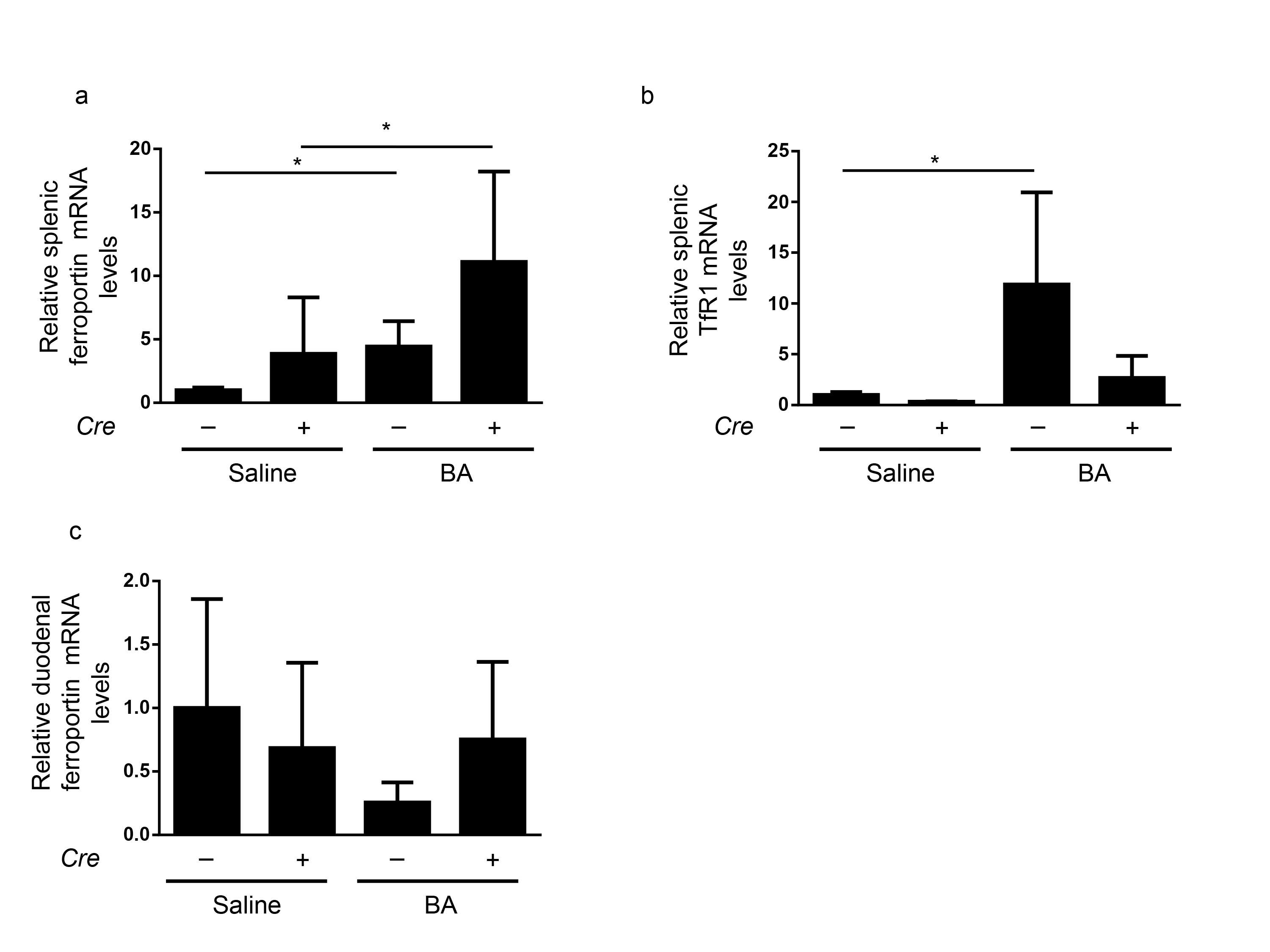

Supplement: Supplementary file 7 — Spleen and duodenum mRNA levels in Alk3fl/fl and Alk3fl/fl;Alb-Cre mice 14 days after BA challenge. (a) Relative splenic ferroportin mRNA levels from Alk3fl/fl and Alk3fl/fl; Alb-Cre 14 days after heat-killed Brucella abortus (BA) injection (*P = 0.02: Alk3fl/fl injected with saline [n = 4] vs Alk3fl/fl injected with BA [n = 5]; *P = 0.03: Alk3fl/fl; Alb-Cre injected with saline [n = 4] vs Alk3fl/fl; Alb-Cre injected with BA [n = 4]. (b) Relative splenic TfR1 mRNA levels (*P = 0.01: Alk3fl/fl injected with saline [n = 4] vs Alk3fl/fl injected with BA [n = 6]). (c) Relative duodenal ferroportin mRNA levels. The relative CT method was used to normalize the levels of target transcripts to 18S rRNA levels. (TIFF 103 kb) [file 12899_2018_37_MOESM7_ESM.tif]

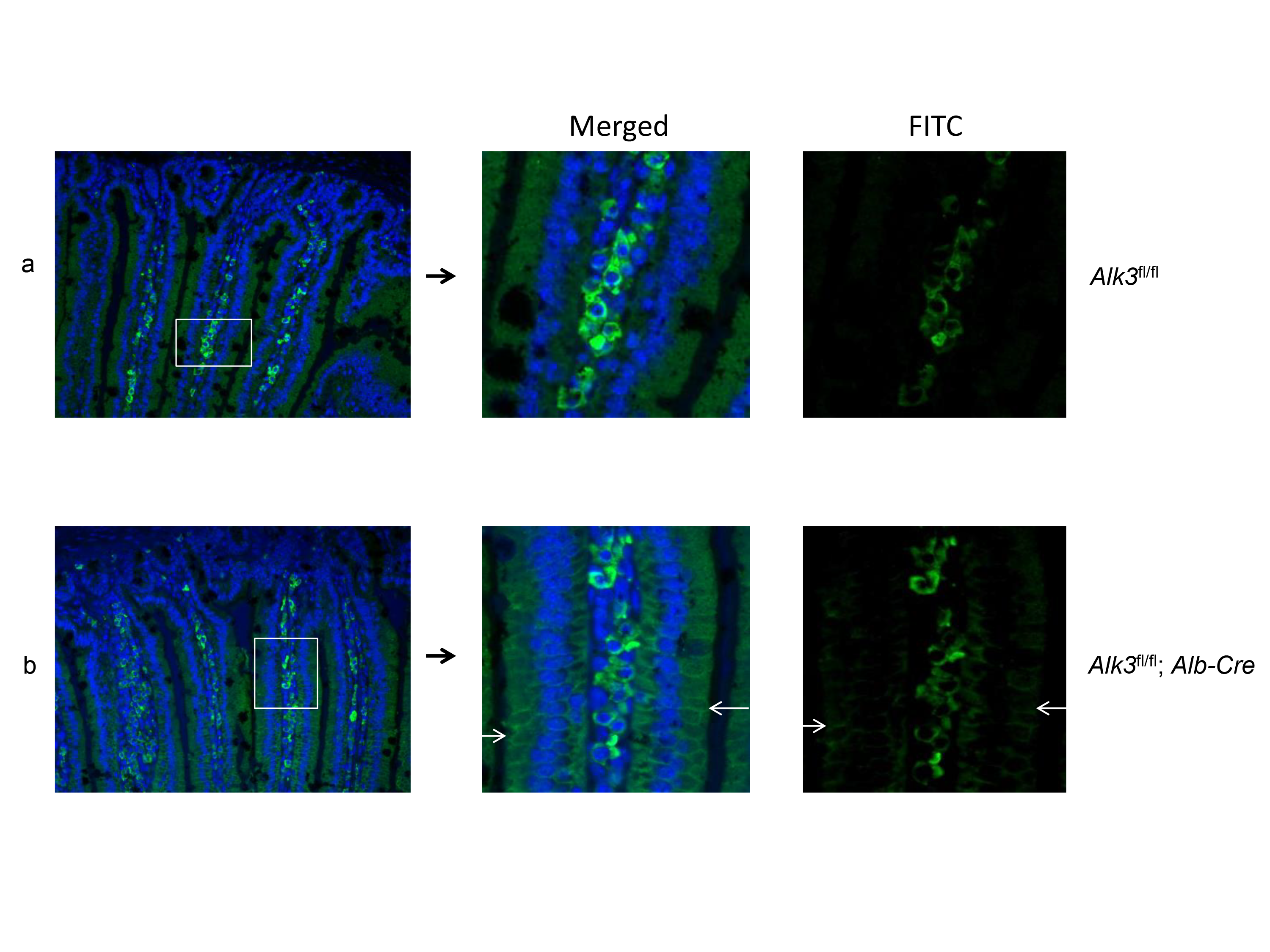

Supplement: Supplementary file 8 — Immunofluorescence staining of ferroportin in the duodenum of Alk3fl/fl and Alk3fl/fl; Alb-Cre mice. Ferroportin immunofluorescence staining of formalin fixed paraffin sections of the villosities of the duodenum with 20 times magnification. (Panel a) Control mice with nuclear (DAPI) and ferroportin (FITC) staining of a duodenal section. Cutout images merged (nuclear and ferroportin) and ferroportin (FITC) alone. (Panel b) Duodenal section of hepatocyte-specific Alk3 deficient mice with cutout images merged (nuclear and ferroportin) and ferroportin (FITC) alone. White arrows highlight specific FPN staining. (TIFF 3601 kb) [file 12899_2018_37_MOESM8_ESM.tif]

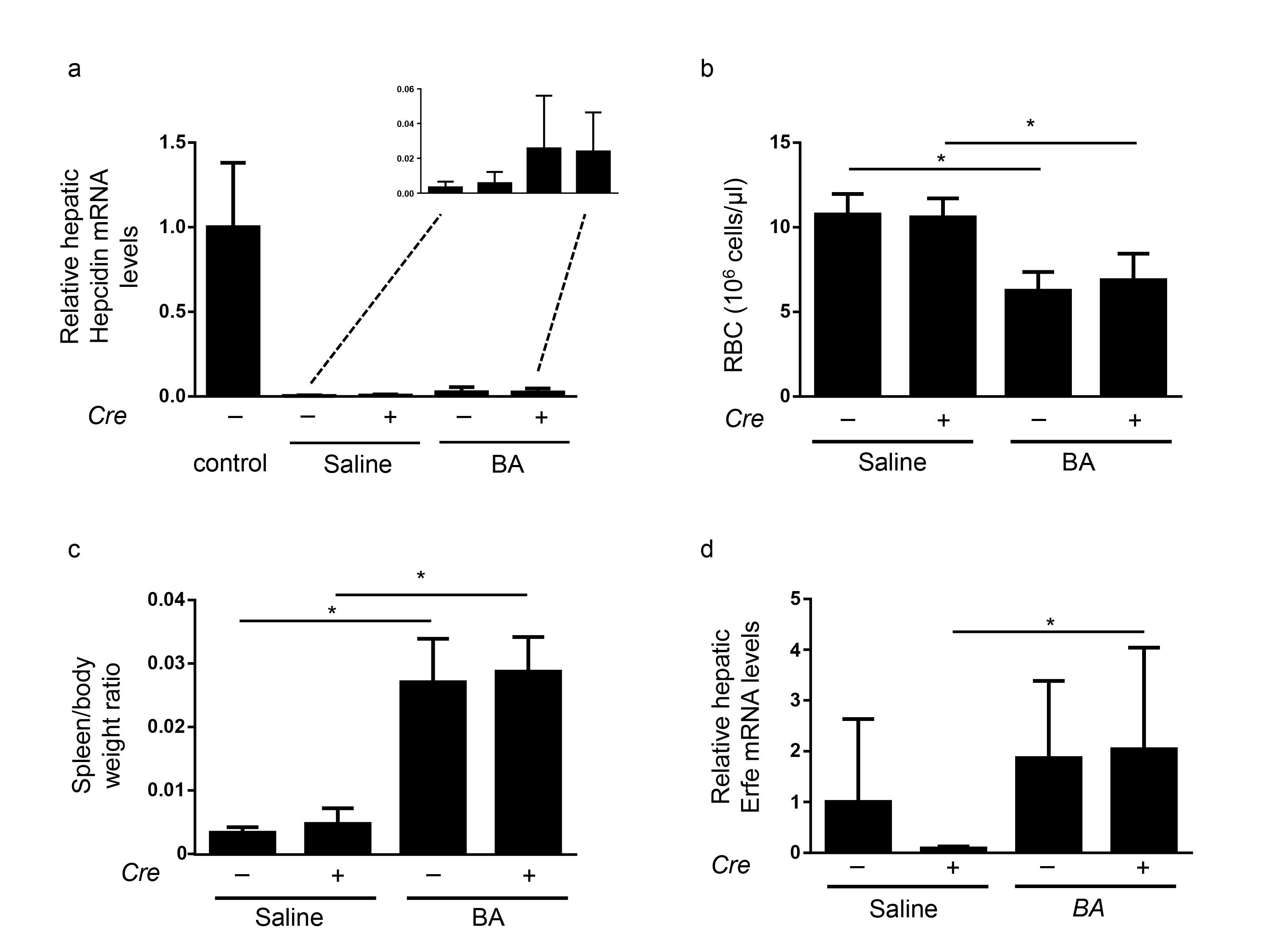

Supplement: Supplementary file 10 — Hepatic hepcidin mRNA levels, red blood cell count, spleen to bodyweight ratio, and hepatic erfe mRNA levels of Alk3fl/fl; Alb-Cre mice and Alk3fl/fl mice. (a) Relative hepatic hepcidin mRNA levels compared to control mice fed a regular diet. The relative CT method was used to normalize the levels of target transcripts to 18S mRNA levels. (b) Red blood cell count from Alk3fl/fl and Alk3fl/fl; Alb-Cre mice 14 days after heat-killed Brucella abortus (BA) injection (*P = 0.01: Alk3fl/fl injected with saline [n = 4] vs Alk3fl/fl injected with BA [n = 6]; *P = 0.02: Alk3fl/fl; Alb-Cre injected with saline [n = 4] vs Alk3fl/fl; Alb-Cre injected with BA [n = 6]). (c) Spleen to bodyweight ratio in Alk3fl/fl and Alk3fl/fl; Alb-Cre mice 14 days after heat-killed Brucella abortus (BA) injection (*P = 0.01: Alk3fl/fl injected with saline [n = 4] vs Alk3fl/fl injected with BA [n = 6]; *P = 0.01: Alk3fl/fl; Alb-Cre injected with saline [n = 4] vs Alk3fl/fl; Alb-Cre injected with BA [n = 6]). (d) Relative hepatic erfe mRNA levels of Alk3fl/fl and Alk3fl/fl; Alb-Cre mice 14 days after heat-killed Brucella abortus (BA) injection (*P = 0.01: Alk3fl/fl; Alb-Cre injected with saline [n = 4] vs Alk3fl/fl; Alb-Cre injected with BA [n = 5]). (TIFF 129 kb) [file 12899_2018_37_MOESM10_ESM.tif]
