# Supplementary material for: Deficiency of the BMP Type I receptor ALK3 partly protects mice from anemia of inflammation
Source: BMC Physiol. 2018 Feb 27;18:3. doi: 10.1186/s12899-018-0037-z (PMC6389079; doi:10.1186/s12899-018-0037-z)
Supplement: Supplementary file 2 — Table S1. Semi-quantitative real-time PCR primer. (DOCX 15 kb) [file 12899_2018_37_MOESM2_ESM.docx]

**Table S1. Semi-quantitative real-time PCR primer.**

| **18S rRNA forward** [1] | **5'-CGGCTACCACTCCAAGGAA-3'** |
| --- | --- |
| **18S rRNA reverse** | **5'-GCTGGAATTACCGCGGCT-3'** |
| **Mouse Hepcidin forward** [1] | **5`-AAGCAGGGCAGACATTGCGAT-3’** |
| **Mouse Hepcidin reverse** | **5`-CAGGATGTGGCTCTAGGCTATGT-3’** |
| **Mouse IL-6 forward** [2] | **5’-AACGATGATGCACTTGCAGA-3’** |
| **Mouse IL-6 reverse** | **5’-TGGTACTCCAGAAGACCAGAGG-3’** |
| **Mouse Irp1 forward** [2] | **5’-TGGGCACAGATTCACACACG-3’** |
| **Mouse Irp1 reward** | **5’-TGTGCAGCTAGTGATGGCAG-3’** |
| **Mouse Irp2 forward** [2] | **5’-TGGAATACCGGCAATGGTGG-3’** |
| **Mouse Irp2 reward** | **5’-AGGACCCGTATTGAGTAAGGC-3’** |
| **Mouse HO-1 forward** [3] | **5’-AAGCCGAGAATGCTGAGTTCA-3’** |
| **Mouse HO-1 reverse** | **5’-GCCGTGTAGATATGGTACAAGGA-3’** |
| **Mouse TNF-α forward** [2] | **5’-AATGGCCTCCCTCTCATCAG-3’** |
| **Mouse TNF-α reverse** | **5’-GCTACGACGTGGGCTACAGG-3’** |
| **Mouse ALK3 TaqMan Gene expression assay** [1] | **Bmpr1a- Mm00477650_m1 Bmpr1a** |
| **Mouse-Ferroportin forward** [4] | **5’-CTCTGTCAGCCTGCTGTTTG-3’** |
| **Mouse-Ferroportin reverse** | **5’-TCAGGATTTGGGGCCAAGATG-3’** |
| **Mouse SAA1 forward** [5] | **5’-** **AGTCTGGGGTGCTGAGAAAA-3’** |
| **Mouse SAA1 reverse** | **5’-** **ATGTCTGTTGGCTTCCTGGT-3’** |
| **Mouse Erfe forward [5]** | **5’- ATG-GGG-CTG-GAG-AAC-AGC-3’** |
| **Mouse Erfe reverse** | **5’- TGG-CAT-TGT-CCA-AGA-AGA-CA-3’** |
| **Mouse TfR1 forward** [1] | **5’- GGAAGACTCTGCTTTGCAGCTAT-3’** |
| **Mouse TfR1 reverse** | **5’-** **GCCCAGGTAGCCCATCATGA-3’** |
