# Supplementary material for: Deficiency of the BMP Type I receptor ALK3 partly protects mice from anemia of inflammation
Source: BMC Physiol. 2018 Feb 27;18:3. doi: 10.1186/s12899-018-0037-z (PMC6389079; doi:10.1186/s12899-018-0037-z)
Supplement: Supplementary file 9 — Supporting material and references. (DOCX 14 kb) [file 12899_2018_37_MOESM9_ESM.docx]

**Additional file 9:**

**Supporting material**

**Immunohistochemistry**

Paraffin- embedded tissue sections were deparaffinized and rehydrated. Heat-mediated antigen retrieval was mediated with Tris/EDTA buffer, pH8. Sections were blocked in PBS with 1% BSA and 10% FCS for 1h and afterwards incubated with anti-MTP1/FPN (dilution 1:50; Cat. No. MTP11-A; Alpha Diagnostics) at 4°C overnight. After three washing steps, sections were incubated with rabbit-anti-FITC (dilution 1:1000; Sigma-Aldrich) containing DAPI. Autofluorescence was reduced with 10% Soudan Black B and sections were mounted with Eukitt® (Sigma-Aldrich). Sections were imaged with an Olympus BX50 microscope and documented with Cell^F software (both Olympus, Hamburg, Germany).

**References**

1. Steinbicker AU, Bartnikas TB, Lohmeyer LK, Leyton P, Mayeur C, Kao SM, et al. Perturbation of hepcidin expression by BMP type I receptor deletion induces iron overload in mice. Blood. 2011;118: 4224-4230.

2. Cui W, Taub DD, Gardner K. qPrimerDepot: a primer database for quantitative real time PCR. Nucleic Acids Res. 2006;35: D805-D809.

3. Steinbicker AU, Sachidanandan C, Vonner AJ, Yusuf RZ, Deng DY, Lai CS, et al. Inhibition of bone morphogenetic protein signaling attenuates anemia associated with inflammation. Blood. 2011;117: 4915-4923.

4. Ramey G, Deschemin J, Durel B, Canonne-Hergaux F, Nicolas G, Vaulont S. Hepcidin targets ferroportin for degradation in hepatocytes. Haematologica. 2009;95: 501-504.

5. Kautz L, Jung G, Valore EV, Rivella S, Nemeth E, Ganz T. Identification of erythroferrone as an erythroid regulator of iron metabolism. Nat Genet. 2014;46: 678-684.
